# The Impact of Immediate and Delayed Rewards on Task-Switching Performance

**DOI:** 10.3390/brainsci15020100

**Published:** 2025-01-21

**Authors:** Guang Zhao, Huijun Wang, Rongtao Wu, Zixin Zhao, Shiyi Li, Qiang Wang, Hong-Jin Sun

**Affiliations:** 1 Faculty of Psychology, Tianjin Normal University, Tianjin 300387, China; 15340705354@163.com (H.W.); wurongtao0331@163.com (R.W.); 19861257085@163.com (Z.Z.); elevenny@hotmail.com (S.L.); wangqiang113@gmail.com (Q.W.); 2 Department of Psychology, Neuroscience and Behaviour, McMaster University, Hamilton, ON L8S 4K1, Canada; sunhong@mcmaster.ca

**Keywords:** task switching, switch costs, immediate reward, delayed reward, cognitive control

## Abstract

Background/Objectives: Switching between different tasks incurs switch costs. Previous research has demonstrated that rewards can enhance performance in cognitive tasks. However, prior studies have primarily focused on the overall improvement in cognitive task performance, with limited research on how different types of rewards function under various task conditions. This study aims to investigate the distinct effects of immediate and delayed rewards on cognitive task performance in different task conditions (repeated trials and task-switching trials) and to explore the underlying neural mechanisms, particularly focusing on how rewards influence attention allocation during the concurrent processing of multiple cues. Methods: This study recruited 27 college students (average age 19 years old, 10 males and 17 females). A cue-based task-switching paradigm incorporating immediate and delayed rewards was employed. The study examined the effects of immediate and delayed rewards on cognitive task performance in repeated trials and task-switching trials. Event-related potentials (ERPs) were recorded to investigate the neural mechanisms underlying reward effects on attention allocation. Results: Behavioral results indicated that immediate rewards significantly enhanced performance in repeated trials compared to delayed rewards. In contrast, no significant difference between immediate and delayed rewards was observed in task-switching trials. ERP results showed that immediate rewards induced a larger P300 amplitude than delayed rewards under the task repetition condition. No P300 difference was found between immediate and delayed rewards under the task-switching condition. Conclusions: The findings suggest that rewards enhance task performance by optimizing the allocation of attention to the ongoing task when multiple cues are processed concurrently. When additional resources are required to process task-related cues, there may be insufficient remaining capacity to effectively process reward cues, which could be essential for the optimal completion of the task. These results support the Expected Value of Control (EVC) theory in task-switching scenarios.

## 1. Introduction

In the course of our everyday activities, individuals are required to switch frequently from one task to another, by which their efficiency at work was reduced. The ability that one has to switch quickly and effectively among tasks largely depends on one’s own cognitive control and cognitive flexibility. In the literature, cognitive control, which enables individuals to prioritize on the specific task, refers to the crucial ability to regulate attention and behavior in order to keep one’s attention on the target when confronted with multiple tasks [1]. Cognitive control is a goal-oriented process where individuals adapt their behavior by flexibly allocating cognitive resources in response to a continuously changing environment [1]. Cognitive flexibility refers to the ability to adapt one’s thoughts and behaviors to new environments, tasks, or changing situations. This capacity allows individuals to adjust rapidly in response to a dynamic environment, enhancing their ability to navigate through unforeseen circumstances, which is crucial for effectively managing unexpected events [2]. Cognitive control and flexibility play essential roles in task switching, as they constitute the core mechanisms by which individuals manage tasks. Understanding these cognitive regulation processes is of significant importance, particularly in complex environments. Numerous researchers have extensively explored cognitive control and cognitive flexibility through task-switching experiments, in which subjects are required to perform at least two tasks and switch flexibly among them [3,4,5,6,7,8]. This revealed a broad cognitive cost when individuals switch between tasks, commonly referring to the switch costs [4,5,8,9,10,11,12].

Research on switch costs has consistently shown that more cognitive resources are required when individuals switch between tasks [4,5,13,14,15,16]. Rewards, as external motivational factors, have been found to enhance individuals’ cognitive resources engagement with tasks, thereby promoting behavioral performance [17,18,19,20,21]. The expected value of control (EVC) theory offers a framework for understanding how rewards affect the allocation of cognitive resources during cognitive tasks [22]. The EVC theory suggests that greater effort costs in a cognitive task are represented by higher cognitive engagement; and these costs can be mitigated by anticipated rewards. It takes into account people’s decision-making, believing that rewards and task performance in the human decision-making process are factors considered in cost–benefit trade-offs, and people tend to choose tasks with higher rewards or lower error rates [23]. Typically, rewards escalate as a function of task performance, which itself tends to improve with increased cognitive engagement. The EVC for cognitive engagement is determined by the net difference between its rewarding benefits and effort costs that are involved during the task performance. The optimal cognitive resource investment is thus the one that maximizes the EVC. As rewards escalate, the expected benefit rises accordingly, driving individuals to increase their cognitive investment to optimize the EVC, consequently enhancing their performance on cognitive tasks [19]. That is to say, rewards enhance task performance by increasing cognitive engagement in the task. In the study by Umemoto and Holroyd [24], the impact of rewards on specific tasks during task switching was also explored. Participants were asked to switch between two tasks, one of which provided rewards (i.e., the reinforced task), while the other did not (the non-reinforced task). The results showed that the switch cost for the non-reinforced task was greater, especially in terms of error rate. This indicates that rewards may improve task performance by increasing top-down control over the reinforced task.

However, in addition to directly manipulating reward magnitude, previous researchers have also discovered the existence of temporal or delayed discounting [25,26,27,28,29]. This means that the subjective value of a reward decreases as time passes—the later the reward is received, the lower the individual’s valuation of its value. Rong [30] studied the impact of rewards on cognitive tasks by manipulating the timing of reward receipt (immediate reward, delayed reward, no reward). The additional reward under the immediate reward condition will be redeemed immediately after the experiment ends, while it will be issued three months later under the delayed reward condition. The results showed that under reward conditions, task performance was better than under neutral conditions, with stronger neural activities under immediate rewards than delayed rewards and neutral conditions. The result provides substantial evidence for the EVC theory. Compared with immediate rewards, delayed rewards have time discounts, and the subjective value of rewards decreases with the increase in time delay. Therefore, the expectation of delayed rewards will reduce cognitive investment in specific tasks, thereby affecting behavioral performance.

The EVC theory provides insights into how reward expectations influence the allocation of cognitive resources in tasks. In addition, researchers have proposed a cognitive control model based on EVC to explain and predict behavioral adjustments in cognitive control tasks, including task switching and reward dependency [31]. In real-world scenarios, frequent task switching is often necessary to achieve goals. Reward expectations might affect the efficiency of switching between different tasks. The expectation of rewards could play a significant role in enhancing cognitive flexibility [32,33,34] and proactive task encoding [35]. For example, Shen and Chun [34] examined task-switching performance under four conditions: reward increase, maintaining high reward, reward decrease, and maintaining low reward. The results showed that the increased reward led to faster response times, and this reward facilitation was more pronounced in the switching task than the repetition task. Moreover, lower task-switching costs were observed when rewards at a high level or decreased condition compared to rewards remained a low level, suggesting that high-value rewards and the dynamic changes in rewards can enhance cognitive flexibility for tasks. Hall-McMaster [35] also reached the same conclusion that reward expectations prompt individuals to switch more fluidly between tasks, thereby enhancing task performance. The study manipulated high and low rewards based on the task-switching paradigm and found that the anticipation of high rewards could bolster proactive encoding of task rules, leading to improved cognitive task performance. Moreover, the reward anticipation was linked to increased neural segregation among task rules, optimizing flexible cognitive control processes. Enhanced neural segregation reduces interference between tasks, allowing for more efficient task switching and better overall performance.

Building on the EVC theory and these findings, the present study aims to further investigate the neural and cognitive mechanisms behind how reward expectations influence task-switching performance. Specifically, we seek to understand how immediate versus delayed rewards differentially affect the cognitive processes involved in task-switching. These processes are essential for comprehending the impact of rewards on task performance. To achieve this goal, we utilized a cue-based task-switching paradigm, where a cue is used to indicate the reward and task conditions. Both immediate and delayed reward types were introduced, and electroencephalograms (EEGs) were recorded. This high temporal resolution provides an accurate method for measuring the timing of attentional and cognitive control processes. In paradigms where cues are used as reward signals, Cue-P3 is an ERP component commonly associated with the evaluation process of reward cues [36]. Its response is consistently enhanced for salient stimuli [37], with particularly pronounced responses to reward cues [38,39]. This indicates that the Cue-P3 is highly sensitive to the motivational significance of stimuli, especially those associated with potential rewards, and it provides valuable insights into how individuals process reward-related cues. In the present study, which compares the effects of immediate versus delayed rewards, we specifically focused on analyzing the Cue-P3 component to gain an accurate understanding of neural mechanisms and delve into how individuals’ expectations of reward influence task performance when switching between different tasks.

Based on logic of task-switching costs and reward-promoting cognitive task performance, we would expect greater task performance in repetition trials compared to switch trials, and superior performance in the immediate reward condition compared to the delayed reward ones. On this basis, our primary focus is task-switching costs under varied reward conditions. Integrating the aforementioned studies, we believe there are two different possibilities. First, drawing insights from Hall-McMaster’s [35] findings, where rewards enhance cognitive flexibility and streamline task-switching efficiency, we hypothesized that the switching cost under the immediate reward conditions would be diminished compared to that under the delayed reward conditions. Second, according to the EVC theory, rewards enhance task performance by increasing cognitive engagement, leading individuals to prioritize the task itself. The switching cost under immediate reward conditions is not expected to significantly decrease compared to that under the delayed reward conditions in the present study. In repetition trials, where cognitive demands are lower and effort costs are reduced, ample cognitive resources can be allocated to assess the impact of rewards, thus leading to better performance under immediate reward conditions compared to delayed reward conditions. Conversely, in switch trials characterized by increased task complexity, more cognitive resources would be allocated to maximize EVC for better task completion, thereby reducing their focus on reward expectations. At this point, the impact of reward expectations on task execution is limited, making the switching cost more pronounced under immediate reward conditions compared to delayed reward conditions. By elucidating the mechanisms through which rewards affect attention allocation and cognitive engagement under different task conditions, our study aims to provide support for enhancing cognitive flexibility and work efficiency. We anticipate that the results of this study may help to optimize learning and working environments, as they offer insights into how to effectively utilize reward systems to improve cognitive performance in complex and dynamic task contexts.

## 2. Materials and Methods

### 2.1. Participants

The sample size for this study was determined based on the effects of the behavioral difference between immediate and delayed reward conditions in the studies of Mason [40]. This study used an average effect size (Cohen’s d) of 0.56, a power analysis using G ∗ Power [41] indicated that a minimum sample size of 20 participants was required to achieve 0.8 power, with an *α* of 0.05 for testing. A total of 27 participants were recruited from the author’s university (average age = 19.4 years, aged from 18 to 24 years) to participate in this experiment. One participant was excluded from further analysis due to electrode damage during the data collection phase, rendering their data unusable. The actual sample size meets the requirements. They volunteered to take part in the study in exchange for a remuneration of RMB 25. All participants were right-handed and had normal or corrected-to-normal vision. The research protocol received approval from the Ethics Committee of the Department of Psychology of the author’s institution. Informed consent was obtained from all participants prior to the experiment.

### 2.2. Materials and Design

**Figure 1:**
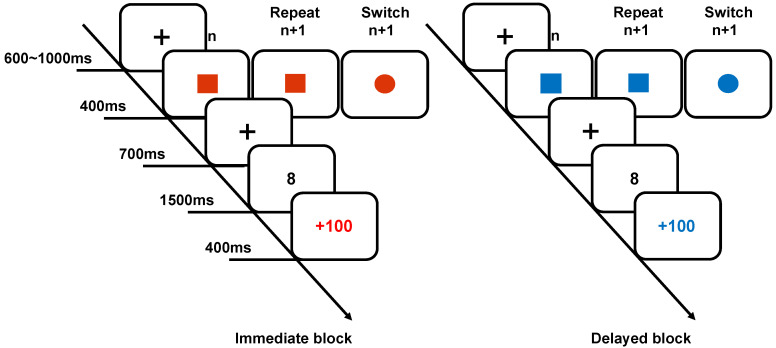
Trial procedure for different blocks. Stimulus and procedure in immediate reward (**left**) and delayed reward (**right**). Colors represent different rewards; shapes represent different tasks. Upon completion of an immediate reward block, participants are immediately issued their reward. In contrast, rewards for the delayed reward blocks will be disbursed six months later.

The experimental procedure was programmed using E-Prime 3.0 and presented on a 19-inch monitor with a resolution of 1024 × 768 pixels and a refresh rate of 60 Hz. Participants were seated 60 cm away from the monitor in a soundproof, well-lit room and instructed to remain as still as possible while responding using the keys (Q, W, O, P) on the keyboard. The experiment utilized a task-switching paradigm, where participants responded to numbers following visual cues, which were square or circular shapes (166 × 155 pixels) displayed in blue or red, corresponding to different reward types. The target numbers were presented in the Microsoft YaHei font, size 36, against a white background. As shown in Figure 1, each trial began with a central fixation point on a white background for 600–1000 ms, followed by a cue screen for 400 ms. After a 700 ms interval, a number appeared for the participants to judge. For square cues, participants assessed the parity of the number (Q for odd, W for even); for circular cues, they evaluated the magnitude (O for <5, P for >5). Feedback was provided after each response, and block totals were displayed with feedback color corresponding to the cue screen color. Participants were advised that a red cue indicated an immediate reward, which would be granted in cash upon completion of each block. Given that scores for each block fluctuated between 5000 and 6000, each participant received a RMB 5 reward for each immediate reward block, accumulating to a total of RMB 15. A blue cue represented a delayed reward, with rewards for the delayed blocks to be distributed six months later. The reward conditions represented by different colors were balanced across participants. To ensure consistency in the number of trials for task repetitions and switches, this study utilized a pseudo-randomized approach to present stimuli in a predetermined sequence. Specifically, in each reward condition, we arranged four orders of stimulus presentation (for example, AABBABBA/BBABBAAA/ABBAAABB/BAAABBAB). These four orders were randomly presented in each block to prevent participants from being able to predict the upcoming task. Ultimately, each block comprised 32 trials of task repetition and 31 trials of task switching, with the initial trial of each block being excluded as a filler and not subjected to analysis. In total, there were approximately 93 trials in each condition. The entire experiment lasted about 30 min. After the experiment, participants received a cash reward of RMB 15 (RMB 5 for each immediate reward block). Six months later, they received the remaining RMB 15.

## 3. EEG Recording and Data Analysis

EEG data were recorded using a 64-channel NeuroScan system (sampling rate: 1000 Hz), which is manufactured by Compumedics Neuroscan (5015 West WT Harris Blvd, Suite E, Charlotte, NC 28269, USA), a company based in the United States, with electrodes placed according to the 10–20 system and impedance kept below 5 kΩ. Additional electrodes monitored eye movements. The Cz electrode served as an online reference, with bilateral mastoids (M1 and M2) re-referenced for offline analysis. EEG data were processed using the EEGLAB 2023 toolbox (a widely used open-source MATLAB2022b toolbox for the analysis of EEG data) in MATLAB: the sampling rate was reduced to 500 Hz [4,30,42,43], artifacts were removed using independent component analysis (ICA), and data were band-pass filtered from 0.01 to 40 Hz [30,35,44] with a notch filter at 48–52 Hz. An artifact rejection criterion of ±80 μV [45] was applied, and ERP analysis was conducted within a −200 to 800 ms window, using the 200 ms pre-stimulus as baseline. Data were manually inspected to eliminate noise, and averages were computed across trials and participants for final ERP results. We focused on the P3 component in the ERP activity during the cue period to analyze how reward cues influence attention allocation under different task conditions.

Statistical analyses were conducted using jamovi2.5.6 (https://www.jamovi.org, accessed on 6 July 2024). Given that our experiment employed a within-subjects design, repeated measures ANOVA was deemed appropriate to examine the effects of the experimental independent variables on these two key dependent variables. For the reaction time data, we first excluded trials with errors and those with reaction times less than 300 ms. Subsequently, we performed a 2 (task repetition, task switching) × 2 (immediate reward, delayed reward) repeated measures ANOVA on the reaction time results. Similarly, for the accuracy rate results, we also conducted a 2 (task repetition, task switching) × 2 (immediate reward, delayed reward) repeated measures ANOVA. For EEG data, we carefully examined the EEG components elicited by cues. Drawing upon prior research and the observed outcomes in this study, electrodes positioned at CP1, CP3, CP5, CPz, CP2, CP4, and CP6 [30,44,46] on the parietal cortex were selected. A temporal window ranging from 350 to 470 ms was chosen to investigate the average P300 component across four conditions using a 2 (Task Repeat, Task Switch) × 2 (Immediate Reward, Delayed Reward) repeated measures of ANOVAs.

## 4. Results

### 4.1. Behavioral Results

**Figure 2:**
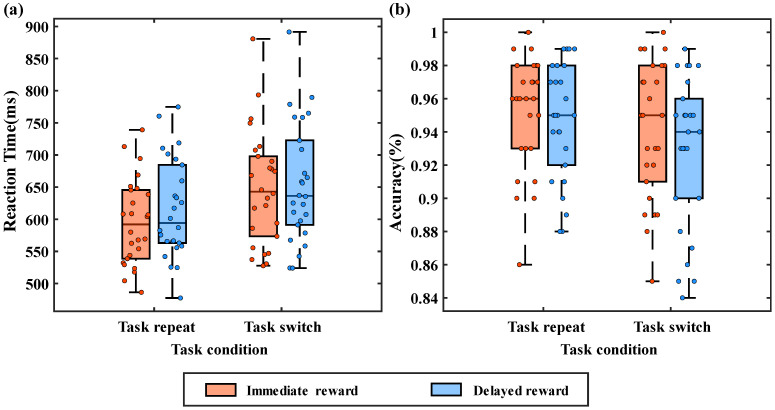
Behavioral performance as a function of reward and task condition. (**a**) Reaction time in four conditions and the scatter plot of reaction time for each participant; (**b**) accuracy under four conditions. Red represents immediate rewards, and blue represents delayed rewards.

**Figure 3:**
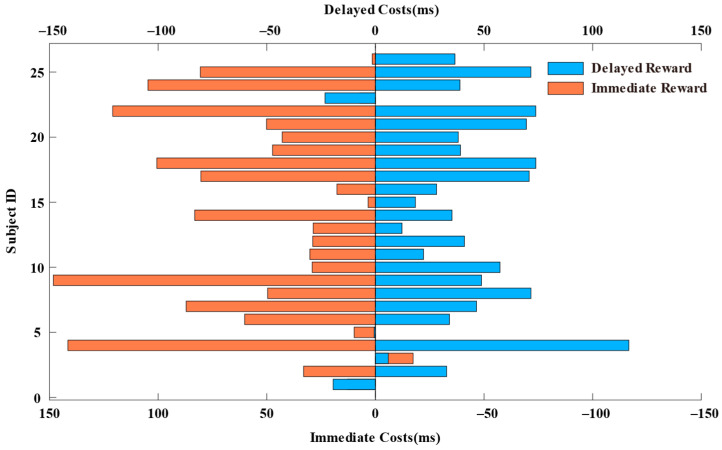
Switch costs in different reward conditions. We subtracted the reaction time for task repetition from the reaction time for task switching, thereby obtaining the switching cost for each participant under the conditions of immediate reward (**left**) and delayed reward (**right**). The bottom *x*-axis represents the coordinates of the switching cost for immediate rewards, with 0 ms to the left indicating positive values and to the right indicating negative values. The top *x*-axis represents the coordinates of the switching cost for delayed rewards, with 0 ms to the right indicating positive values and to the left indicating negative values.

Upon primary examination, 1 participant was excluded due to electrode malfunction, leaving 26 participants for subsequent analysis. Incorrect and missing and trials with RT less than 300 ms were excluded from the RT analyses. To examine whether the difficulty levels of the two tasks are consistent and whether task difficulty affects our experimental results, we first conducted a repeated-measures ANOVA on reaction time (RT) using task type (parity task, size task), task condition (task repetition, task switching), and reward condition (immediate reward, delayed reward) as within-subject factors. The results revealed significant main effects for task condition, with *F*(1, 25) = 44.481, *p* < 0.001, *η_p_*^2^ = 0.640. Additionally, the main effect of reward condition was significant, *F*(1, 25) = 6.561, *p* = 0.007, *η_p_*^2^ = 0.208. Furthermore, task type also showed a significant main effect, *F*(1, 25) = 23.997, *p* < 0.001, *η_p_*^2^ = 0.490. However, significant interactions were only found between task condition and reward condition, *F*(1, 25) = 4.770, *p* = 0.039, *η_p_*^2^ = 0.160, while the interactions between task type and task condition, and between task type and reward condition, were not significant. Therefore, we believe that although there are differences in the difficulty levels of the two tasks, the task difficulty does not affect our analysis of the switching costs under different reward conditions. Subsequently, we will only analyze and discuss the two factors of task condition and reward condition. Repeated measures ANOVAs were performed on RT and accuracy using task (repeat, switch) and reward condition (immediate, delayed). For mean RT (see Figure 2a), the ANOVAs revealed a significant main effect of task, *F*(1, 25) = 45.40, *p* < 0.001, *η_p_*^2^ = 0.645. Participants showed significantly faster responses during task repetition trials compared to task-switching trials, consistent with previous research highlighting evident switch costs during task switching [4,5,8,12]. The main effect of the reward is significant, *F*(1, 25) = 6.66, *p* = 0.016, *η_p_*^2^ = 0.210. Specifically, responses were marginally faster in blocks featuring immediate rewards, consistent with the literature suggesting immediate rewards enhance cognitive task performance. More importantly, a significant interaction was observed, *F*(1, 25) = 4.60, *p* = 0.042, *η_p_*^2^ = 0.155. Further analysis revealed that in task repetition trials, the effect of the reward was significant, *t* (25) = −3.74, *p* < 0.001, Cohen’s d = −0.734, indicating faster response times under the immediate reward conditions compared to the delayed reward conditions. However, in task-switching trials, the effect of the reward was not significant, *t* (25) = −1.11, *p* = 0.277, Cohen’s d = −0.218, suggesting no significant difference in response times between immediate and delayed reward conditions. To further explore how rewards impact task-switching across different conditions, we calculated and analyzed task-switching costs under different reward conditions (as shown in Figure 3, which describes the distribution of switch costs for each participant under immediate and delayed reward conditions). We conducted a paired-sample *t*-test on the switch costs under the two reward conditions. The results showed a significant difference between immediate and delayed rewards, *t* (25) = 2.15, *p* = 0.04, Cohen’s d = 0.421, indicating greater switch costs under the immediate reward condition (53 ms) than the delayed reward condition (40 ms). These behavioral results preliminarily suggest that the improvement in task performance due to rewards is not achieved by enhancing the efficiency of switching between tasks.

Similarly, for accuracy rates (as shown in Figure 2b), a significant main effect of task sequence emerged, *F*(1, 25) = 6.48, *p* < 0.05, *η_p_*^2^ = 0.206, indicating higher accuracy rates during task repetition trials. However, the main effect of the reward approached marginal significance, *F*(1, 25) = 3.04, *p* = 0.093, *η_p_*^2^ = 0.109, the interaction effect was not significant, *F*(1, 25) = 0.73, *p* = 0.4, *η_p_*^2^ = 0.029. The accuracy results reflected evident switch costs, consistent with the reaction time analyses. Nevertheless, no significant differences were observed regarding reward type and interaction effects.

### 4.2. EEG Results

**Figure 4:**
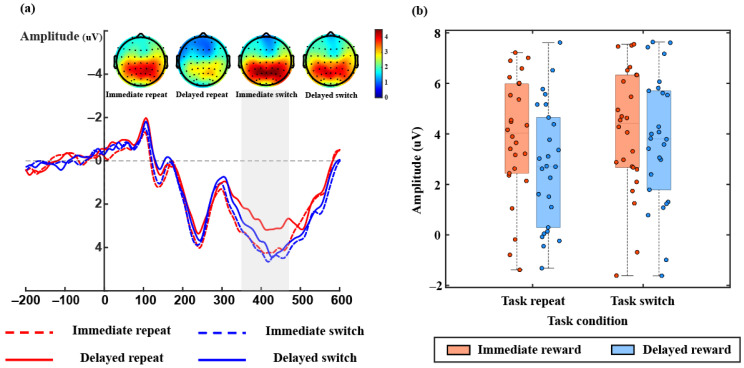
ERP results in all conditions. (**a**) ERP waveform and topographic map results in different conditions, representing the activation of brain regions in different conditions within the P300 time window; (**b**) ERP result boxplots and scatter plots corresponding to each condition within the P300 time window.

Figure 4a shows the average ERP waveforms under the four conditions along with the corresponding topographic maps, while Figure 4b presents bar graphs of mean ERP amplitudes corresponding to different conditions. During the P300 component, the ANOVA revealed a main effect of task, *F*(1, 25) = 11.09, *p* = 0.003, *η_p_*^2^ = 0.307, indicating that task-switching elicited larger P300 components compared to task repetition. Additionally, there was a significant main effect of reward, *F*(1, 25) = 8.16, *p* = 0.008, *η_p_*^2^ = 0.246, indicating that immediate reward conditions elicited larger P300 components. Moreover, a significant interaction effect was observed, *F*(1, 25) = 4.81, *p* = 0.038, *η_p_*^2^ = 0.161. Further analysis compared the impact of rewards on neural activity across different task environments. During task repeat trials, an immediate reward elicited stronger P300 activity compared to a delayed reward, with significant differences observed, *t* (25) = −3.67, *p* = 0.001, Cohen’s d = −0.719. However, during task switch trials, there was no difference in the P300 component elicited by immediate versus delayed reward, *t* (25) = −1.11, *p* = 0.276, Cohen’s d = −0.219. As P300 reflects the process of task set updates and attention allocation [30,42,47,48,49,50,51], our results support the hypothesis that the improvement in task performance from immediate and delayed rewards is not due to increased efficiency in switching between tasks. Instead, it is because rewards promote individuals to continuously evaluate the expected value of control at various cognitive control levels based on environmental changes and behavioral rewards, selecting the optimal control level to maximize rewards.

## 5. Discussion

This study used a cue-based task-switching paradigm to investigate immediate and delayed rewards on task performance across various task sequences. Consistent with previous research, we observed the typical task-switching cost [4,5,8,9,10,11,12], where participants exhibited slower reaction times and lower accuracy during task switches compared to task repetitions. Furthermore, the ERP results indicated a stronger P300 amplitude during task switches, suggesting the updating of task-relevant information or task sets [42,47,48,49,50,51]. We also identified a main effect of reward, with task performance under immediate reward conditions being significantly better than under delayed reward conditions [41,52,53,54]. More importantly, we found a significant interaction between the task sequence and the reward condition. This is a result we have found in our research that has not been mentioned before. As for accuracy, the lack of interaction might be due to the relative simplicity of the experimental task, leading to consistently high average accuracy rates across all four conditions. This may obscure any effects of reward type and interaction due to a ceiling effect.

### 5.1. Task Switching Demands More Cognitive Resources

Switching between different tasks often involves a range of cognitive costs, known as switch costs. The current results are consistent with the literature [4,5,13,14,15,16]. Participants exhibited slower reaction times and lower accuracy during task switching, regardless of the reward condition, indicating typical task-switching costs. These costs arise because task switching demands more cognitive resources. Several theories explain switch costs. The task set reconfiguration theory [5,11,13] suggests that inhibiting the old task set and activating the new task set requires cognitive resources, resulting in switch costs. The task set inertia theory [3,8] emphasizes the interference effects of the preceding task on the subsequent task. Alternatively, the competition hypothesis [7,8,55,56] posits that executing the current task competes with rules associated with the prior task, increasing cognitive load and requiring additional stabilization time, thus causing task-switching costs. Despite their different focuses, these theories all examine the relationship between old and new task sets, elucidating the mechanisms underpinning switch costs from distinct perspectives. The ERP results regarding task sequences also indicated that during cue locking, task switching elicited a larger P300 amplitude compared to task repetition. This suggests that during the task preparation phase, participants actively updated the task set or task-related information to better complete the task [42,47,48,49,50,51].

### 5.2. Expected Rewards Affect Cognitive Task Performance

The expectation of rewards can affect task performance, regardless of manipulating the value of rewards or the timing of reward allocation. In our study, we manipulated the timing of reward distribution between blocks. Our results showed that task performance was better under immediate reward conditions, with faster reaction times and higher accuracy. These findings are consistent with previous research, indicating that reward expectations significantly affect cognitive task performance [25,26,28,29]. For example, Rong [30] demonstrated that the enhancements in task performance were significantly more pronounced under conditions of immediate reward anticipation. This was achieved by varying the immediacy and delay of rewards between trials. This result aligns with research that directly manipulates the reward value [35,36]. This is because the motivational effect of reward expectation is influenced by temporal or delay discounting, which diminishes with time delay [40,52,53,54]. Therefore, the subjective value of immediate rewards is higher. These findings support the viewpoint of the (EVC) theory. Additionally, neural activity results also provide evidence for the EVC theory. During cue locking, there were significant differences in the P300 component under different reward conditions. This result is consistent with Rong [30], which revealed a more robust P300 component in immediate reward scenarios, indicating heightened attentional allocation to cues with greater value salience (Immediate Reward > Delayed Reward > No Reward) during the early stages of processing. This suggests that participants allocated more attentional resources to high-value cues to better complete the task, suggesting the transformation of motivational salience into cognitive control and effort allocation [19,20,56,57].

### 5.3. Rewards Varies Under Different Tasks

Aside from the results consistent with previous research, we focus on discussing the interaction between task type and reward type. Our behavioral results indicated a significant interaction between these variables, with a higher switching cost under immediate reward conditions compared to delayed reward. The impact of rewards varies under different task conditions. Hall-McMaster [35] also adopted the task-switching paradigm to explore the impact of different rewards on tasks by manipulating the value of rewards and the obtained consistent main effect results. However, their research did not find any interaction, suggesting no significant difference in switch costs under different reward conditions. Our results are inconsistent with this finding. This discrepancy might be due to the presentation of the cues. Hall-McMaster [35] presented reward and task cues separately, with the task cues presented sequentially after the reward cue, while in our study reward cues and task cues were presented simultaneously on the display. When both reward and task cues are presented sequentially, participants would have sufficient time and resources to process those cues in a separate way. Thus, both cues could be fully processed and have an impact on subsequent task performance. However, when those two types of cues are presented simultaneously, limited cognitive resources prevent participants from processing different cues simultaneously, by which participants have to adjust their strategies to obtain more rewards. According to the EVC theory, the acquisition of rewards depends on task performance. During task repetition trials, participants are more familiar with the task, allowing them to anticipate higher reward levels. In such scenarios, individuals can maximize EVC by investing greater cognitive resources and effort costs into the current task.

Additionally, both immediate and delayed rewards, as well as high and low rewards, generally conformed to the EVC theory, and yielded similar results across different studies [30]. Participants perceive immediate rewards as having a higher subjective value. They would allocate more cognitive resources and better performance in immediate reward blocks compared to delayed reward conditions. As shown in the results of the P300 component in this study, immediate rewards induced stronger neural activity than delayed rewards during task repetition. This may be due to the fact that performing task repetition trials is relatively simple and does not require much cognitive resource investment. Moreover, in the delayed reward block, participants are aware that they cannot receive rewards immediately, according to the EVC theory, which could lead to a decrease in motivation, and they may exert less cognitive effort. As a result, the smallest EEG amplitude is induced under the delayed reward task repetition condition. Conversely, for task-switching trials facing new situations, participants must relearn and adjust their cognitive strategies to task requirements. They ought to invest more cognitive resources to adapt to these new tasks. Here, effectively executing new tasks or successfully adapting to new task requirements may become more crucial in obtaining rewards. Thus, participants invested more cognitive resources in processing task-related information and paid less attention to the impact of rewards, resulting in no difference in the impact of rewards on task performance. The P300 component results during task switching in this study provide evidence that there is no difference between ERP activity induced by immediate or delayed rewards. Therefore, immediate rewards have a better promotion on task repetition trials than delayed rewards, while there is no difference between immediate and delayed rewards on task-switching trials, resulting in higher switch costs under the immediate rewards than the delayed rewards condition.

## 6. Limitations and Future Directions

This study investigated the differential effects of immediate and delayed rewards on cognitive task performance under different task conditions and their neural mechanisms. While we have obtained meaningful results, there are several limitations that future research can address. Firstly, previous research has shown that experimental intervals or task preparation times can significantly influence task-switching costs [5,58,59]. Longer task intervals can reduce task-switching costs because participants have more time to prepare for the upcoming task. Building on our findings, future studies could extend the task preparation time, allowing participants more time to prepare, which may yield different results. Additionally, task preparation time could be strategically manipulated as an influencing factor, and stimulus intervals could be systematically varied to explore the dynamic effects of rewards under different task conditions. Secondly, in order to explore the relationship between reaction time results and EEG amplitude, we conducted correlation analysis on behavioral and EEG data. No significant correlations were found under all the four conditions. This may be due to the small sample size, which may bring some limitations. Future research should consider increasing the sample size and the number of trials to ensure that the results are more reliable and generalizable. In addition, the EVC theory takes into account people’s decision-making, that is, people can decide for themselves what tasks to perform. In this study, participants carried out the corresponding tasks according to the cue prompts. Future research could allow participants to choose tasks autonomously for investigation.

## 7. Conclusions

This study adopted the task-switching paradigm to manipulate immediate and delayed rewards between blocks to explore the impact of different rewards on different task sequences and their neural mechanisms. The results showed a higher switching cost under the immediate reward condition than that under the delayed reward conditions. The ERP results during the cue stage showed that the difference in P300 wave amplitude between immediate and delayed rewards was greater during task repetition than that during task switching. The results suggested that when multiple cues processed simultaneously, participants would have to adjust their cognitive strategies to optimize task performance and obtain more rewards.

## Data Availability

The raw data supporting the conclusions of this article will be made available by the authors on request.
